# Response planning during question-answering: does deciding what to say involve deciding how to say it?

**DOI:** 10.3758/s13423-023-02382-3

**Published:** 2023-09-22

**Authors:** Ruth E. Corps, Martin J. Pickering

**Affiliations:** 1 Psychology of Language Department, Max Planck Institute for Psycholinguistics, Max Planck Institute for Psycholinguistics, Nijmegen, The Netherlands; 2 Department of Psychology, University of Edinburgh, 7 George Square, Edinburgh, EH8 9JZ UK

**Keywords:** Language production, Question-answering, Response planning

## Abstract

To answer a question, speakers must determine their response and formulate it in words. But do they decide on a response before formulation, or do they formulate different potential answers before selecting one? We addressed this issue in a verbal question-answering experiment. Participants answered questions more quickly when they had one potential answer (e.g., *Which tourist attraction in Paris is very tall?*) than when they had multiple potential answers (e.g., *What is the name of a Shakespeare play*?). Participants also answered more quickly when the set of potential answers were on average short rather than long, regardless of whether there was only one or multiple potential answers. Thus, participants were not affected by the linguistic complexity of unselected but plausible answers. These findings suggest that participants select a single answer before formulation.

## Introduction

To answer a question, speakers must determine their response and formulate it in words. But how are these processes related? Do they decide on a response before formulation, or do they formulate different potential responses before selecting one? To address this issue, we conducted a question-answering experiment in which questions had only one potential answer (e.g., *Which tourist attraction in Paris is very tall?*) or multiple potential answers (e.g., *What is the name of a Shakespeare play*?). These potential answers varied in their linguistic complexity (e.g., *Macbeth, Romeo and Juliet, A Midsummer Night’s Dream*), and so we could determine whether the complexity of unselected, but plausible, answers affected processing difficulty.

Language production involves conceptualization (i.e., message preparation), formulation (i.e., linguistic encoding), and articulation (Levelt et al., 1999). Selecting an answer is an aspect of conceptualization, but this answer then has to be formulated—the words have to be retrieved from the lexicon, assigned to a grammatical structure, and converted into phonological representations. Do speakers select one answer when conceptualizing, and then pass it onto formulation? Or do they consider more than one answer when conceptualizing, and select among these alternatives when formulating?

When a question has one answer, speakers will of course consider just that answer (unless they make an error). But when the question has many answers, we assume that listeners will often consider different answers, which will cause additional processing difficulty. Consistent with this assumption, research has shown that people find it harder to retrieve answers when they know more about a particular concept (e.g., Anderson, 1974, 1981; Lewis & Anderson, 1976; Radvansky et al., 2017). For example, participants are slower to recognize a studied statement, such as *The hippie is in the park*, if they have also studied *The hippie is in the church* than if they have studied no other statements about *the hippie*. This result is known as the *fan effect* because retrieval and recognition involve searching for the target among all the facts known about a particular concept, and the time taken to recognize a particular statement increases with the number of statements “fanning out” from the concept.

As a result, speakers may find it harder to answer questions with multiple answers because they have to search through a pool of potential answers before selecting the final one. But the complexity of the answers in the pool may also affect the ease of question-answering. This issue relates to the cascade of activation in the lexicon, which has been central to debates within language production. This debate has typically focused on whether speakers who are naming an object activate just the phonology of the name (serial activation; e.g., Levelt et al., 1991), or whether they also activate the phonology of semantically related words (cascaded activation; e.g., Peterson & Savoy, 1998). For example, Levelt et al. (1991) found that picture-naming times (e.g., to name a sheep) were not affected when pictures were preceded by words phonologically related (e.g., *goal*) to semantic associates (e.g., *goat*) of the picture name, suggesting that unselected words activated at the semantic level were not converted into phonological representations. In support of cascaded processing, Peterson and Savoy (1998) found that participants were quicker to name a visual target word when it was phonologically related to the picture’s secondary name (e.g., *soda* for a picture whose dominant name was *couch* and secondary name was *sofa*) than when they named unrelated control words.

Most theories now agree that production involves at least some cascade of information through the language production system (e.g., Roelofs, 2008; Roelofs & Ferreira, 2019; Strijkers & Costa, 2016). As a result, we might assume that speakers will formulate different answers to a question, only selecting a single answer at the end of formulation. If this is the case, then answer selection should be affected by the linguistic properties of unselected answers. Question-answering typically occurs in conversation (i.e., one person asks a question and another responds), and research has shown that interlocutors in conversation produce utterances with little gap between their contributions (around 200 ms; Stivers et al., 2009). Selecting an answer at the end of formulation would allow speakers to begin formulating potential answers as soon as they are activated at the conceptual level, thus facilitating production and enabling the speaker to respond quickly. We refer to this possibility as the *selection-after-formulation* account. Consistent with this account, blend errors suggest that alternative messages can be considered and partly formulated (e.g., Harley, 1984).

However, previous studies typically required production of a single message (or a clearly determined message).^1^ But during question-answering, the speaker must typically consider unrelated messages. Furthermore, studies supporting cascaded processing have typically focused on single word production, where participants name pictures in the presence of distractors. Formulating multiple potential responses in single-word production is likely to be less cognitively demanding than formulating multiple potential responses during question-answering, where responses likely involve phrases or sentences. Furthermore, speakers typically begin planning an answer while still comprehending the speaker’s question (e.g., Bögels et al., 2015; Corps et al., 2018). Planning while comprehending is cognitively demanding (e.g., Fairs et al., 2018), and so speakers may select a single answer during conceptualization to minimize cognitive demands. If this is the case, speakers select a single answer before formulating it, and make a (final) decision about the message without converting that message into words. As a result, answer times should be unaffected by the linguistic properties of other unselected, but plausible, answers because they are not formulated. We refer to this possibility as the *selection-before-formulation* account.

A study by Ferreira and Swets (2002; Experiment 1) is somewhat consistent with this account. They found that participants took longer to produce answers to sums (e.g., 21 + 23) both when the adding tens of the sum was difficult rather than easy and when adding the ones was difficult rather than easy. Thus, the difficulty of both of the tens and the ones contributed to initiation times, suggesting participants began speaking only once they had selected an answer and knew both parts of the sum. However, the arithmetic problems had only one correct answer, and so this study tells us about the relationship between planning (conceptualization and formulation) and speaking, but not about the relationship between conceptualization and formulation.

We tested between the selection-after-formulation and selection-before-formulation accounts using a verbal question-answering task, in which we manipulated the ease of selecting an answer by manipulating the constraint of questions. Some questions constrained responses to a particular answer (constraining questions; e.g., *Which tourist attraction in Paris is very tall?*), so that participants would typically linguistically encode only one message as their answer. Other questions did not constrain responses to a particular answer (unconstraining questions; e.g., *What is the name of a Shakespeare play*?), so that participants were able to linguistically encode multiple potential messages as alternative answers (see Table 1). Participants should answer more quickly when questions are constraining rather than unconstraining because they have fewer concepts to search in memory before selecting one.

**Table 1:** Example stimuli for the four stimuli conditions

Question constraint	Answer length	Question
Constraining	Short	Which creature lives in the sea and has eight tentacles?
	Long	Which tourist attraction in Paris is very tall?
Unconstraining	Short	What is the name of an animal that has two ears?
	Long	What is the name of a Shakespeare play?

To determine whether participants formulated unselected, but plausible, answers, we also manipulated the length of the potential answers so that they were either short or long (i.e., multiword) phrases. Note that participants could choose how they responded, but the single answer provided by the majority of participants in a pretest to select the stimuli was short or long in the constraining conditions, and the *set* of potential answers provided in the pretest were on average short or long in the unconstraining conditions. Research suggests that it takes longer to initiate longer than shorter utterances (e.g., Ferreira, 1991; Smith & Wheeldon, 1999), and so it should be easier to formulate a short rather than a long answer.

The relationship between question constraint and answer length is critical for determining between the selection-before-formulation and selection-after-formulation accounts. If participants formulate multiple potential answers, as predicted by the selection-after-formulation account, then we expect an interaction between question constraint and answer length. In particular, we expect stronger effects of answer length when the question is unconstraining rather than constraining because speakers will tend to activate and formulate a larger set of linguistically complex items. In contrast, if speakers select a single answer during conceptualization, as predicted by the selection-before-formulation account, then participants should be slower to answer unconstraining than constraining questions, regardless of whether the set of potential answers is long or short because they will formulate only one answer. Note that we present the selection-before-formulation and selection-after-formulation accounts as two alternatives, but it is also possible that different situations elicit different production strategies. We return to this issue in the Discussion.

We first conducted two pilot studies (Pilot 1 with 40 native English speakers; Pilot 2 with 41 nonnative English speakers), which showed that participants were faster to answer constraining (Pilot 1, *M* = 647 ms; Pilot 2, *M* = 1,177 ms) than unconstraining questions (Pilot 1, *M* = 1,279 ms; pilot 2, *M* = 1,816 ms). They were also faster when to-be-prepared answers were short (Pilot 1, *M* = 834 ms; Pilot 2, *M* = 1,335 ms) rather than long (Pilot 1, *M* = 1,086 ms; Pilot 2, *M* = 1,643 ms). In both pilot studies, we found a pattern suggesting there was no interaction between these two factors, supporting a selection-before-formulation account. However, these studies had at least two limitations. First, we considered unconstraining questions to be those that elicited different answers across participants, with the assumption that participants would consider (and retrieve) multiple different answers for these questions. But it is possible that each individual participant considered only one plausible answer. In the experiment we report, we ensured that unconstraining questions elicited different answers within participants. Second, unconstraining questions in the pilot studies tended to be opinion-based (e.g., *What is your favourite book*?) while constraining questions were fact-based (e.g., *What colour is broccoli*?). Given these limitations, we do not use the results of these pilot studies to draw conclusions about the relationship between selection and formulation in question-answering. Instead, we use them to derive predictions about expected effect sizes so we can compute Bayes factors for our effects, especially since the selection-before-formulation account predicts a null interaction. We also calculated our power for detecting an interaction, if it were to exist (see Results).

### Method

#### Participants

We selected 40 participants (38 females, two males; *M*_age_ = 27.45 years) for analysis from a sample of 50 native English speakers who were recruited from Prolific Academic and participated in exchange for £1.25. We discarded data from 10 participants, either because their audio responses we not clearly audible (one participant) or because they listened to the questions using headphones, which made it impossible for us to determine answer times. All participants resided in the United Kingdom and had a minimum 90% satisfactory completion rate from previous assignments. Participants had no known speaking, reading, or hearing impairments. This sample size was based on our pilot studies, and previous question-answering experiments (e.g., Corps et al., 2018).

#### Materials

We selected 60 questions (15 per condition) using an online norming task, in which 20 native English speakers from the same Prolific Academic population (18 females, two males; *M*_age_ = 26.10 years) were randomly assigned to one of two stimulus lists. Participants in each list were visually presented with 100 questions and told: “You will see a question displayed on-screen. Sometimes the question will have only one answer, while other times there will be multiple potential answers. You have 10 seconds to provide as many answers to the question as you can.” Participants typed as many answers as they could into the text box provided before the ten seconds time-out. The experiment was administered online using jsPsych (Version 6.0.5; De Leeuw, 2015).

We assessed question constraint by determining the number of different answers participants produced for each question. Questions in the constraining condition tended to elicit one answer, while those in the unconstraining condition tended to elicit multiple different answers, *F*(1, 56) = 171.28, *p* < .001 (see Table 2), and the short and the long answer conditions did not differ in the number of answers they elicited, *F*(1, 56) = 3.81, *p* = .06. There was an interaction between question constraint and answer length, *F*(1, 56) = 4.98, *p* = .03, such that unconstraining-long questions elicited fewer answers than unconstraining-short questions, *t*(1, 17) = −2.13, *p* = .048. This pattern makes sense, given that participants would have less time to produce many different long answers than many different short answers, simply because each answer takes time to produce. We return to this interaction in the Data Analysis section. The two constraining conditions did not differ, *t*(1, 17) = 0.71, *p* = .48.

**Table 2:** Means (and standard deviations) of the number of different answers, answer word length, and question duration (ms) for stimuli

Question constraint	Answer length	Number of answers^a^	Answer word length^b^	Question difficulty	Question duration
Constraining	Short	1.03 (0.07)	1.04 (0.09)	6.50 (0.76)	3640 (952)
	Long	1.05 (0.08)	2.23 (0.58)	6.55 (0.61)	3587 (1062)
Unconstraining	Short	2.14 (0.50)	1.02 (0.04)	6.81 (0.32)	2813 (860)
	Long	1.83 (0.18)	2.23 (0.51)	6.74 (0.27)	2978 (671)

^a^ Mean number of different answers participants provided to questions in the online pretest

^b^ Mean word length of all answers provided to a particular question in the online pretest

Participants in the pretest provided answers with a mean word length between 1.00 and 1.30 for stimuli in the short conditions, and between 1.62 and 4.00 for stimuli in the long conditions (see Table 2 for means). Answers in the short condition were significantly shorter than those in the long condition, *F*(1, 56) = 144.01, *p* < .001, but there was no difference in the length of answers for the constraining and unconstraining questions, *F*(1, 56) = 0.01, *p* = .91, and no interaction between question constraint and answer length, *F*(1, 56) = 0.02, *p* = .89.

We assessed the difficulty of the questions in a separate online pretest, in which 10 native English speakers from the same Prolific Academic population (nine females, one male; *M*_age_ = 27.67 years) rated the difficulty of answering each question on a scale of 1 (*very difficult to answer*) to 7 (*very easy to answer*). We calculated the average difficulty rating for each question. Questions were rated as easy to answer (an average rating of 6.65). Importantly, there was no difference in the difficulty of answers in the four conditions (all *p*s > .06; see Table 2).

Questions were recorded by a native English female speaker who was instructed to read utterances as though “you are asking a question and expecting a response.” Recordings were between 1,525 and 6,177 ms (see Table 2). Questions were significantly longer in the constraining than the unconstraining condition, *F*(1, 56) = 9.60, *p* = .003. This difference may influence any effects of question constraint. In particular, previous research suggests that longer questions elicit earlier answers (Corps et al., 2018) and so participants may answer constraining questions more quickly simply because they are longer, rather than because there is only one candidate answer. This explanation seems unlikely, given that our pilot experiments showed effects of question constraint when conditions were matched for average duration. However, we included question duration in our model to ensure any effect of question constraint was not influenced by question duration. Importantly, question duration did not differ in the two answer length conditions, *F*(1, 56) = 0.06, *p* = .81, and there was no interaction between question constraint and answer length, *F*(1, 56) = 0.22, *p* = .63.

#### Procedure

We administered the experiment online. Recent research suggests that although data collected online may be noisier than in the laboratory, with longer tails in the distribution, onset latencies can be measured with good accuracy (Fairs & Strijkers, 2021; Stark et al., 2021; Vogt et al., 2021). These studies have also replicated key findings in the speech production literature, such as frequency effects (Fairs & Strijkers, 2021) and cumulative semantic interference effects (Stark et al., 2021).

Stimulus presentation and data recording were controlled by jsPsych. Participants were told that they would be listening to audio stimuli and would have their voice recorded, so they were encouraged to complete the experiment in a quiet environment using their computer speakers. Before beginning the experiment, participants checked their microphone was clearly recording their answers. They read the sentence “This experiment is fun” and then listened to their audio recording to ensure they could clearly hear themselves. If they could not, they were asked to move their microphone closer and create another test recording, ensuring they could hear themselves. 

Participants pressed the spacebar to begin audio playback of the question. A fixation cross (+) appeared 500 ms before question onset, and the fixation cross turned red as audio playback began. Following Corps et al. (2018), participants were instructed to “Answer the question with the word or words that you think are most appropriate as quickly as possible. Do not wait until the speaker has finished the question and has stopped speaking. Instead, you should answer as soon as you expect the speaker to finish the question.” Thus, participants were encouraged to prepare a response as soon as possible (rather than simply wait for the speaker to finish) and articulate it close to the speaker’s turn-end. Participants spoke into their microphone and pressed the space bar after answering the question to begin the next trial. Participants completed four initial practice trials to familiarize themselves with the experimental procedure before they were presented with the 60 experimental stimuli (15 from each condition) in an individually randomized order.

#### Data analysis

Answer times were calculated manually in Praat and were the interval between question end (calculated by determining the question’s duration) and the beginning of the answer, ignoring any nonspeech sounds such as audible in-breaths but including disfluencies (e.g., *uhh*).^2^ Answer times were negative when participants answered before the end of the speaker’s question and positive when they answered after the end. We removed four (0.17%) answer times greater than 10,000 ms, as they were clear outliers. We also removed 51 (2.13%) answers because the participant did not provide an audible response. We then replaced 68 (2.90%) answer times at least 2.5 standard deviations above the by-participant mean and seven (0.30%) answer times at least 2.5 standard deviations below the by-participant mean with the respective cut-off value.

We evaluated the effects of question constraint and answer length on answer times with linear mixed-effects models (Baayen et al., 2008) using the *lmer* function of the *lme4* package (Version 1.1-31; Bates et al., 2022) in RStudio (Version 2022.12.0+353). Answer times were predicted by question constraint (reference level: unconstraining vs. constraining), answer length (reference level: long vs. short), and their interaction. These predictors were contrast coded (−0.5, 0.5) and centered. Since previous research suggests answer times are affected by stimulus duration (e.g., Corps et al., 2018), we also included (centered) question duration as a fixed effect in our analysis. Models fitted using the maximal random effects structure resulted in a singular fit error, likely because including by-participants random effects for question constraint accounted for zero variance. We thus removed this predictor from the random effects structure, and only included by-participants random effects for answer length and its interaction with question constraint.

Note that when we assessed the number of answers participants provided in the pretest, there was an interaction between question constraint and answer length (see Materials section). In particular, unconstraining-long questions elicited fewer answers than unconstraining-short questions, but this was not the case for the constraining questions. This interaction could have occurred because the unconstraining-long questions were more constraining than the unconstraining-short questions. This pattern might attenuate any interaction between question constraint and answer length: Participants might answer unconstraining-long questions more quickly than unconstraining-short questions simply because the former is more constraining than the latter, rather than because they formulate multiple linguistically complex answers. The two constraining conditions did not differ in the number of elicited answers, and so these conditions should not differ on the basis of strength-of-constraint.

But even if the interaction between question constraint and answer length is attenuated by strength-of-constraint, it should still occur under the selection-after-formulation account. Even if the unconstraining-long questions are more constraining than the unconstraining-short questions, participants should still experience more difficulty in the unconstraining-long condition than the unconstraining-short condition because these questions are still unconstraining (and more unconstraining than the constraining questions) and participants will have to formulate multiple linguistically complex answers.

It is much more likely that the interaction in the pretest was a by-product of the experimental procedure rather than differences in constraint, especially since all of our questions were general knowledge and easy to answer. Participants were given ten seconds to produce as many answers as possible, and it will take longer to produce longer answers. As a result, participants would have had more time to produce multiple different short answers than multiple different long answers, creating the impression that unconstraining-short questions were less constraining than unconstraining-long questions when the differences merely reflect the amount of time participants had to provide their answers in the pretest.

But to preview our results, we found no evidence for an interaction between question constraint and answer length. We conducted power analyses to determine whether we had sufficient power to detect the interaction. We also calculated Bayes Factors for all predictors by fitting Bayesian mixed-effects models using the *brms* package (Version 2.18.0; Bürkner, 2018). We calculated Bayes Factors by comparing with the predictor of interest (e.g., question constraint; M1) to a reduced model without this predictor (M0). A Bayes Factor of approximately 1 indicates no evidence in favour of either model. As the Bayes Factor increases over 3, evidence in favor of the M1 strengthens; as the Bayes Factor decreases under 0.3, evidence strengthens in favour of the M0 (e.g., Kass & Raftery, 1995; Lee & Wagenmakers, 2013).

We fitted models using informative priors, based on our expectations from our pilot studies. All priors were set using a normal distribution. We expected response times to average around 900 ms, with some variability, and so for the Intercept we set a prior with a mean of 900 ms and a standard deviation of 200 ms. We expected a negative effect of question constraint (i.e., faster responses for constraining than unconstraining questions) and answer length (i.e., faster responses for short than long answers), but we expected the effect of answer length to be smaller than the effect of question constraint. As a result, we set a prior with a mean of −300 ms and a standard deviation of 200 ms for the effect of question constraint and a prior with a mean of −100 ms and a standard deviation of 200 ms for answer length. We did not expect an interaction between these two predictors, and so we set a prior with a mean of 0 ms and a standard deviation of 200 ms for the interaction coefficient. For the standard deviation parameter, we set a prior with a mean of 0 ms and a standard deviation of 50 ms; for sigma, we set a prior with a mean of 0 ms and a standard deviation of 100 ms. We did not calculate a Bayes Factor for question duration because we included this predictor as a control variable; we were not interested in whether it affected response times.

We first fitted a model that simulated data from the priors, and then visualized the distribution of effects to ensure they matched our expectations. Once we confirmed that the priors seemed plausible, we fitted models with the actual data. Bayes Factors are sensitive to the choice of prior, and so we also conducted a sensitivity analysis (Schad et al., 2022). We kept the same means as defined in our informative priors, but we changed the standard deviation of each parameter. In particular, we defined a range of priors with standard deviations from 300 ms to 1,000 ms, in increments of 100 ms representing increasingly looser priors and increasing uncertainty about the effect size.

For each predictor, we report coefficient estimates (*b*), standard errors (*SE*), and *t* values. We assume that an absolute *t* value of ±1.96 or greater indicates significance at the 0.05 alpha level (Baayen et al., 2008). For the Bayesian analysis, we report the Bayes Factors (BF) from the informative model only and do not report the model outputs, but these can be found on Open Science Framework. We also report whether the BF was consistent across the sensitivity analysis. All analyses scripts and raw data are available (https://osf.io/y42je/).

### Results

**Fig. 1:**
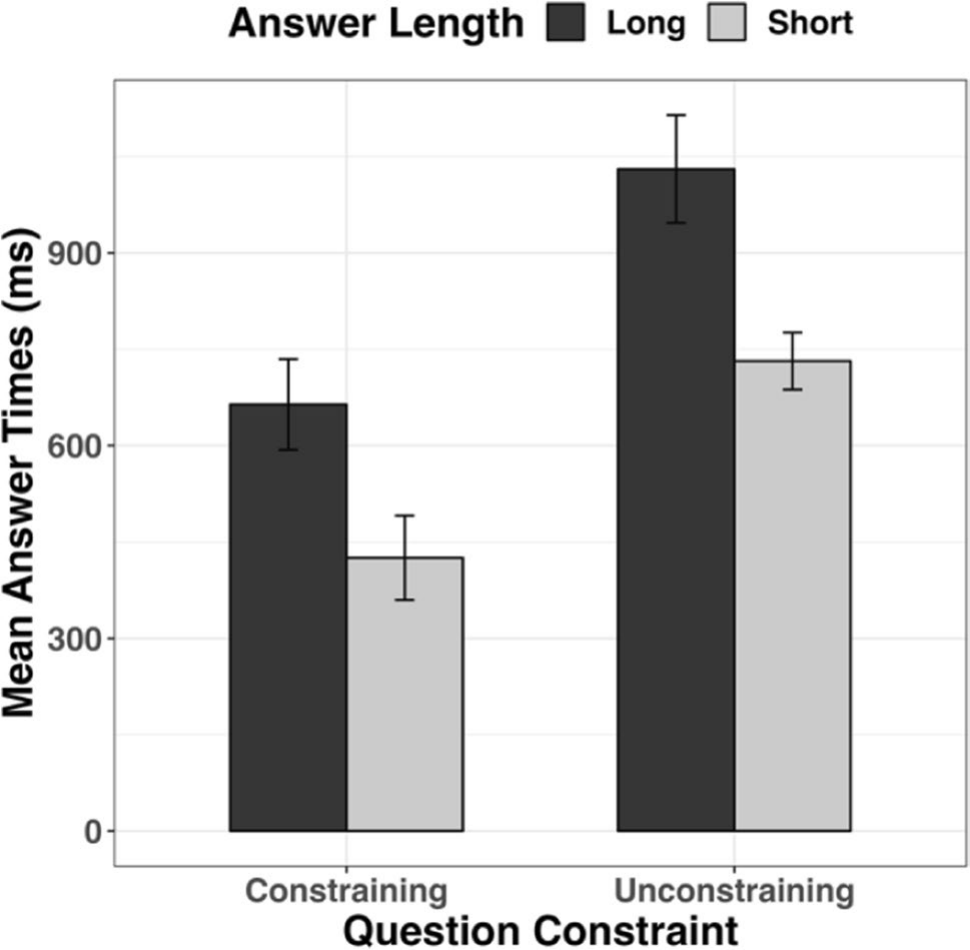
Observed means of answer times for the four conditions. Error bars represent ± 1 standard error from the mean

**Fig. 2:**
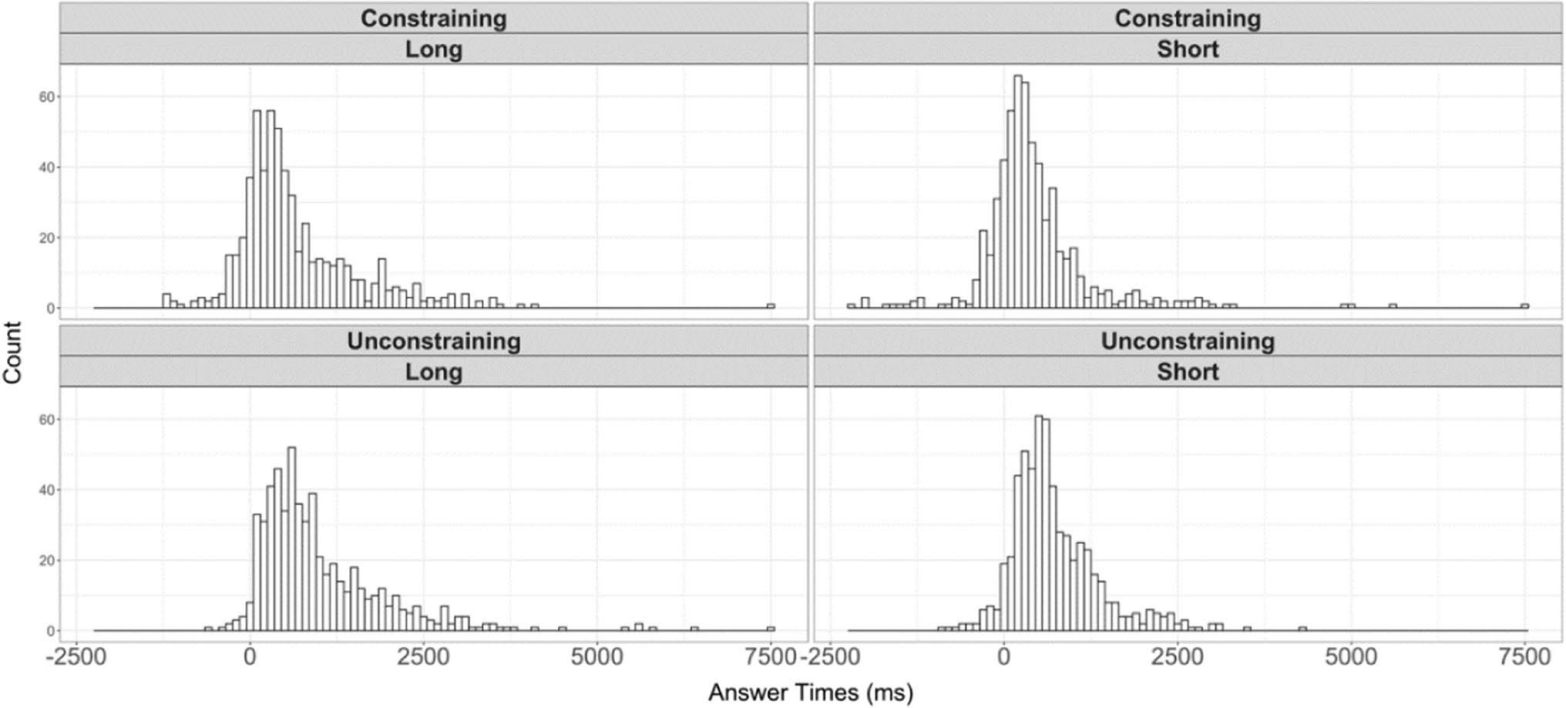
The distribution of answer times for the four conditions. Trials are placed into 100 ms time bins

On average, participants answered 712 ms after the end of the speaker’s question (Fig. 1) and 92% of answers occurred within 2,000 ms of the speaker’s question end (see Fig. 2).

Participants answered more quickly when questions were constraining (*M* = 545 ms) rather than unconstraining (*M* = 879 ms; *b* = −273.62, *SE* = 86.55, *t* = −3.16, BF = 10), suggesting that ease of speaking is affected by ease of retrieving a particular concept from memory. The sensitivity analysis suggested there was evidence for small effect sizes (for models fitted with up to 400 ms standard deviation), but was inconclusive about larger effect sizes (for models fitted with between 500–1,000 ms standard deviation). Participants answered more quickly when to-be-prepared answers were short (*M* = 580 ms) rather than long (*M* = 844 ms; *b* = −277.66, *SE* = 82.87, *t* = −3.35, BF = 188). The BF showed consistent evidence for the alternative hypothesis over the null in the sensitivity analyses (all BFs > 38). There was no effect of question duration (*b* = −77.73, *SE* = 43.44, *t* = −1.79).

Most importantly, there was no interaction between question constraint and answer length (*b* = 68.35, *SE* = 161.84, *t* = 0.42, BF = 0.18). The BF showed consistent evidence for the null hypothesis over the alternative in the sensitivity analyses (all BFs < 0.11). These findings are consistent with a selection-before-formulation account, and suggest that speakers select a single answer before beginning formulation.

To test whether the null interaction was due to a lack of power, we conducted a power simulation using a confirmatory model containing a significant interaction effect. We based the size of the expected interaction on the size of our main effects. In particular, we calculated the effect sizes of our main effects using Cohen’s *d* ([difference in means of the two conditions]/sigma; Rouder et al., 2012). The effect size was 0.39 for the question constraint effect and 0.32 for the answer length effect. Thus, we expected the interaction effect size to be no larger than 0.3, corresponding to a beta coefficient of 273 ms.

We then determined the power to detect this effect. To do so, we created an artificial dataset containing 60 items and 40 participants (i.e., as in the experiment) and added two factors corresponding to our fixed effects (i.e., question constraint and answer length). Next, we created an artificial linear mixed effects model using the simr package (Version 1.0.6; Green & MacLeod, 2016). We estimated the variance in our random effects and the residual variance (sigma) of the model using values from our actual analysis. We did the same for the beta coefficients for the fixed effects, but we set the beta coefficient for the interaction to 273 (corresponding to an effect size of 0.3). We then used this model for a power simulation using the mixedpower package (Version 0.1.0; Kumle et al., 2021). We performed both databased and smallest-effect-sizes-of-interest (SESOIs) simulations, determining SESOIs by reducing all beta coefficients by 15%. We ran our simulation with 40 participants using 5,000 simulations. Power estimates for the fixed effects for databased and SESOI simulations can be found in Table 3. Importantly, we had sufficient power to detect the imagined interaction, even when we reduced the beta coefficient by 15%, suggesting our null effect cannot be attributed to a lack of statistical power. Note that we did not use the pilot data for power analyses because the (null) interactions in these experiments showed trends in the incompatible with either account (i.e., a larger length effect for constraining than unconstraining questions), likely because the stimuli were not as well controlled.

**Table 3:** Power estimates for each of the fixed effects

Fixed effect	Simulation method	Beta coefficient	Power estimate
Question constraint	Databased	−332.94	0.98
	SESOI	−283.00	0.92
Answer length	Databased	−272.95	1.00
	SESOI	−232.01	1.00
Question Constraint × Answer Length	Databased	273.00	0.99
	SESOI	232.05	0.96

Simulations are either based on the actual coefficients (databased) or the smallest effect size of interest (SESOI; 15% reduction in the actual beta coefficient)

Interactions are often smaller than the main effects (and note the much larger *SE* for the interaction), and so we also ran further simulations with smaller effect sizes. In particular, we set effect sizes of 0.25 (corresponding to a beta coefficient of 215 ms) and 0.2 (corresponding to a beta coefficient of 172 ms). We again ran our simulations with 40 participants using 5,000 simulations. We ran databased simulations only because these effect sizes already correspond to our smallest effect sizes of interest (i.e., they are smaller than an effect size of 0.3, which we would expect). For an effect size of 0.25, we had 93% power to detect an effect; for an effect size of 0.2, we had 78% power. Thus, we had sufficient power to detect even smaller effect sizes for the interaction.

## General discussion

In this experiment, we used a verbal question-answering task to investigate whether speakers select a single answer before formulation, or whether they instead formulate different potential answers before selecting one of them. We found that participants answered more quickly when questions constrained responses to a particular answer than when they did not, suggesting they found it harder to retrieve an answer when there were multiple plausible answers. Participants also answered more quickly when the potential answer or answers were on average short rather than long, consistent with research demonstrating that speakers are affected by utterance complexity (e.g., Ferreira, 1991; Smith & Wheeldon, 1999). Importantly, there was no interaction between these two factors and the Bayes Factors strongly supported this null effect.

Our findings are therefore incompatible with a selection-after-formulation account of answer preparation, which claims that speakers select a single answer only after they have formulated different potential answers, thus making a decision about the answer only after they have converted different potential messages into words. Instead, our results are compatible with a selection-before-formulation account, which claims that speakers select a single answer before formulating it, and thus make a decision about the answer without converting the message into words. As a result, our findings suggest that deciding what to say need not involve deciding how to say it.

Our findings are consistent with research demonstrating that participants find it harder to retrieve statements when they know more about a particular concept (e.g., Anderson, 1974). This finding, known as the fan effect, has been consistently demonstrated using tasks where participants study a set of facts and they have to distinguish the facts they studied from those that they did not. The difficulty is thought to occur because the time taken to recognize a particular statement increases with the number of statements “fanning out” from the concept. Our results extend this research to language production, demonstrating that participants find it harder to retrieve an answer for language production when the question has more potential answers. In other words, when there are more answers “fanning out” from the question, participants have more difficulty retrieving a single answer.

This issue is related to the cascade of activation within the lexicon. Our findings suggest that participants consider multiple potential answers at the conceptual level. But participants were unaffected by the linguistic complexity of unselected, but plausible, answers, suggesting participants considered only one answer during formulation. These findings appear inconsistent with studies showing cascaded activation during lexical access, but these studies have primarily been concerned with concepts related to a single message (e.g., Cutting & Ferreira, 1999; Peterson & Savoy, 1998). Additionally, they have typically been concerned with the flow of information between the lexical and phonological levels, and have focused less on the flow of information between the conceptual and lexical levels. Finally, these studies have typically used picture naming, where participants need to produce only a single word.

Participants in our experiment were required to produce multiword answers (usually phrases) in response to a prerecorded speaker’s question, and so our task was likely more cognitively demanding than the picture naming tasks typically used to investigate the cascade of information between lexical and phonological levels. Additionally, research has shown that speakers typically begin planning an answer while still comprehending the speaker’s question (e.g., Bögels et al., 2015; Corps et al., 2018), and planning in this way is cognitively demanding (e.g., Fairs et al., 2018). As a result, speakers may select a single answer during conceptualisation to minimize the cognitive demands of speaking while simultaneously listening. If listeners know *what* they are going to say early, then they can dedicate their processing resources to determining *how* to say that particular response, rather than other responses that may not be produced.

Note that we are not claiming that this selection-before-formulation strategy applies to all types of language production. In some situations, such as in our experiments, formulating multiple answers may be difficult or the speaker may be confident in what they want to say, and so they will allocate their cognitive resources to selecting an answer early. But in other situations, it is possible that the speaker may think about the potential answers to a question (or the potential words they wish to produce from amongst a pool of alternatives), and so they will select an answer late, during formulation. Research suggests that the scope of response preparation is flexible, and affected by factors such as time pressure (e.g., Ferreira & Swets, 2002), the familiarity of lexical items (e.g., Konopka, 2012), or the ease of construction a sentence (e.g., Wagner et al., 2010). It is thus possible that what speakers formulate is also flexible—sometimes they may formulate multiple alternatives, but in other cases they may formulate only one possibility.

In conclusion, we have shown that speakers in a verbal question-answering task select a single answer before formulating this answer, thus deciding what they want to say before considering how they will say it. In particular, participants answered questions more quickly when they had one potential answer (e.g., *Which tourist attraction in Paris is very tall*?) than when they had multiple potential answers (e.g., *What is the name of a Shakespeare play*?). Participants also answered more quickly when the set of potential answers were on average short rather than long, regardless of whether there was only one or multiple potential answers. These findings suggest that participants select a single answer before formulation, and thus they were unaffected by the linguistic complexity of other potential answers.

## Data Availability

All data, analysis code, and materials are available at: https://osf.io/y42je/.

## Footnotes

1 A possible exception is that naming a target picture can be affected by a distractor picture whose name is phonologically related to the target’s name (Navarrete & Costa, 2005). However, it is possible that these effects reflect sequential rather than concurrent naming.

2 Note that participants produced a disfluency on 133 trials, but our results were the same when we excluded these trials from data analysis.
